# Wear particles enhance autophagy through up-regulation of CD147 to promote osteoclastogenesis

**DOI:** 10.22038/IJBMS.2018.29347.7093

**Published:** 2018-08

**Authors:** Baohua Su, Deng Li, Jie Xu, Yingbin Zhang, Zhiqing Cai, Max Daniel Kauther, Ruofan Ma

**Affiliations:** 1 Department of Orthopedics, Sun Yat-sen Memorial Hospital, Sun Yat-sen University, Guangzhou, China, 510120; 2 Department of Orthopedics and Trauma Surgery, University Hospital Essen, University of Duisburg-Essen, Hufelandstrasse 55, 45147 Essen, Germany

**Keywords:** Autophagy, CD147, Osteoclastogenesis, Peri-implant osteolysis, RANKL

## Abstract

**Objective(s):**

The study aimed to uncover the underlying mechanism linking wear particles to osteoclast differentiation, and we explored the effect of titanium particles of different sizes on CD147 expression and autophagy in macrophages.

**Materials and Methods:**

Effects of titanium particles on CD147 and RANKL mRNA were detected by QPCR; protein level of CD147 and Beclin-1 were detected by Western blot; soluble RANKL were detected by ELISA. To determine the effect of CD147 and autophagy, KG-1a cells were transfected with siRNA-CD147 or treated with autophagy inhibitor CQ (chloroquine), and then co-cultured with different sizes of titanium particles.

**Results:**

Our results showed that 0.2-1.2 µm and 1.2-10 µm titanium particles up-regulate CD147 to activate autophagy, which increase the level of soluble RANKL to promote osteoclastogenesis. Suppression of CD147 with siRNA could diminish particle-induced autophagy and soluble RANKL expression. In addition, CQ could dramatically reduce particle-induced soluble RANKL expression.

**Conclusion:**

Our ﬁndings suggested a possible mechanism underlying wear debris-induced osteolysis and identiﬁed CD147 as a potential therapeutic target in aseptic loosening.

## Introduction

It has been well recognized that wear particles derived from implants have the potential to stimulate inflammation and osteoclast differentiation, which increase osteoclastic bone resorption and decrease osteoblastic bone formation. However, the underlying mechanism linking wear particles to osteoclast differentiation remains to be illustrated. Artificial joint replacement (AJR) is considered one of the most important treatment strategies for patients with end-stage joint disease. However, peri-implant osteolysis (PIO) and subsequent aseptic loosening of the implant are frequent complications which restrict the long-term success of the therapy (1). Titanium wear particles and other types of biomaterial wear byproducts derived from the bone-prosthesis interface are recognized to cause PIO, which then initiates aseptic loosening (2, 3). Although the precise mechanism by PIO is not clear, many studies have suggested that over-activation of osteoclasts and inflammatory responses play a critical role in PIO(4-6). In the periprosthetic tissue, wear particles could be recognized and phagocytosed by macrophages (7, 8), which stimulate macrophages, fibroblasts osteoblasts and T lymphocytes to produce high levels of proinflammatory cytokines and chemokines (9-11). Those proinflammatory cytokines can induce osteoclastogenesis and suppress osteoblast function (12, 13). In addition, wear particles also could enhance osteoclastic bone resorptionby increasing the recruitment of osteoclast precursors, differentiation of osteoclast precursors, activation of osteoclasts, and survival of osteoclasts (14-16). Furthermore, a growing number of research indicate the receptor activator of nuclear factor kappa B (RANK) and its ligand (RANKL) are essential for wear particle-induced osteolysis (17-19). RANK is a membrane-bound factor expressed on the surface of macrophages. RANKL could bind to RANK, then activate downstream signaling cascade pathways to promote the differentiation of bone marrow-derived macrophages to multinucleated osteoclasts (20, 21). Therefore, increased osteoclasts lead to bone resorption surrounding the implant.

Autophagy is a lysosomal degradation pathway, which is responsible for degrading and recycling protein aggregates, oxidized lipids, damaged organelles and intracellular pathogens (22, 23). Factors that induce cellular stress, such as hypoxia, accumulation of oxidative stress and caloric restriction, can induce autophagy (24, 25). In recent years, numerous studies show that autophagy is important to osteoclasts and bone resorption *in vitro* and in *vivo *(26-28). Resorptive activity is decreased when the autophagy is suppressed by bafilomycin in osteoclasts (29, 30). An increase in autophagy has been observed during osteoclastrogenesisunder hypoxia environment and increased oxidative stress (31). In addition, pharmacological and genetic inhibition of autophagy could reduce osteoclastogenesis and bone resorption, inhibiting bone loss caused by ovariectomy or glucocorticoid treatment in mice (32). With respect to wear particle-induced osteolysis, study have reported that CoCrMo metal particles stimulated autophagy in osteoblasts and particle-induced osteolysis animal models (33). However, there is limited research focused on the relationship between autophagy and wear particle-induced osteoclastogenesis.

CD147 is also known as Basigin/Leukocyte Activation Antigen M6/EMMPRIN, which is a transmembrane glycoprotein belonging to the immunoglobulin superfamily (34). CD147 is involved in tissue remodeling, cancer progression and the synovial membrane of rheumatoid arthritis patients (35, 36). CD147 could enhance autophagy of HCC cells and favor HCC cell survival under cisplatin treatment (37). In addition, CD147 plays an important role in breast cancer-induced osteolyticlesions (38). In this case, the abnormal expression of CD147 could induce excessive osteoclast differentiation and bone resorption. Furthermore, some studies showed that CD147 could promote the formation of functional osteoclasts (39). Nevertheless, there is no report about the relationship between CD147 and wear particle-induced osteoclastogenesis.

In the present study, we hypothesized that titanium wear particles could increase the expression of CD147, which could activate autophagy in macrophages. Autophagy may promote the expression of RANKL and induce osteoclastogenesis, whichconsequently caused osteolysis. We tested and verified our hypothesis in the human acute myelogenous leukemia cell line KG-1a.

## Materials and Methods

***Cell culture***

The cell line KG-1a (macrophage) was obtained from ATCC, USA; ATCC number: CCL-246^TM^. KG-1a cells were routinely maintained in Iscove’s Modified Dulbecco’s Medium (IMDM, Gibco, Grand Island, NY, USA) containing 20% heat inactivated fetal bovine serum (FBS, Gibco, Grand Island, NY, USA) in a humidified incubator at 37 ^°^C in an atmosphere of 5% CO_2_ (40).

***Preparation of particles***

Commercial pure titanium particles were purchased from Alfa Aesar (Ward Hill, MA, USA; 00681). The preparation of particles were as described previously (40), the particle size was <20 μm, all of the particles were diluted with pure water and filtered by Millipore filter membranes (Billerica, MA, USA) of a series of sizes (pore diameter: 0.2, 1.2, and 10 μm), three ranges of particle size, 0.2–1.2, 1.2–10, and >10 μm were obtained after filtration. Finally, all particles were washed with 70% ethanol for 24 hr at room temperature, then they were dried in a biological drying oven. The dried particles were sterilized with ethylene oxide. 

According to particle weight and density, the concentration of the particles suspended in phosphate buffered saline (PBS) was adjusted to 5×10^8^ /ml. KG-1a cells were seeded in 6-well plates, 5 million cells per well, three ranges of particle (0.2–1.2, 1.2–10, and >10 μm) were added to the corresponding well, the ratio of particle count to cell count was 1000:1, 500:1, 100:1, 10:1, 1:1, 0:1, respectively, shaken gently for more than 15 mins to make the particles and KG-1a cells mix completely, then cultured with a humidified incubator at 37 ^°^C in an atmosphere of 5% CO_2_ (40).

***Treatment with siRNA and Chloroquine (CQ)***

SiRNA-CD147 and negative control siRNA were purchased from GenePharma (Shanghai, China; SG1062). Transfection was performed as described previously (40). KG-1a cells were transfected with siRNA-CD147 or negative control siRNA using Lipofectamine 2000 reagent (Invitrogen, Waltham, MA, USA; 11668019). 24 hrs post-transfection, the cells were incubated with 0.2–1.2 µm (at particle count to cell count ratio of 100:1) or 1.2-10 µm (at particle count to cell count ratio of 1000:1) titanium particles for 24 hr. 

KG-1a cells were treated with 100 µM CQ (Sigma-Alorich, St. Louis, MO, USA; C6628) for 24 hr, and then co-cultured with 0.2–1.2 µm (at particle count to cell count ratio of 100:1) or 1.2-10 µm (at particle count to cell count ratio of 100:1) titanium particles for 24 hr.

***Quantitative real time PCR (qRT-PCR)***

CD147 and RANKL mRNA levels were detected 6 hr after the incubation of particles and KG-1a cells. Quantitative real-time PCR was performed as described previously (40). Total RNA was extracted using TRIzol reagent (Invitrogen, Waltham, MA, USA; 15596026). Reverse transcription was performed with PrimeScript™ RT reagent Kit and gDNA Eraser (Takara, Tokyo, Japan; RR047A) according to the manuscripts. The qRT-PCR was performed on MiniOpticon™ real-time PCR detection instrument (Bio-Rad, Hercules, CA, USA) with GoTaq® qPCR Master Mix ((Promega, Madison, WI, USA) following standard procedures. A housekeeping gene GAPDH was used as an internal control. 

***Western blot***

Protein levels of CD147 and beclin-1 were detected 24 hr after the incubation of particles and KG-1a cells. The treated cells were lysed with RIPA containing proteinase and phosphatase inhibitors (Sigma-Alorich, St. Louis, MO, USA; Y0001009). The protein was quantified with Pierce™ BCA Protein Assay Kit (ThermoFisher, Waltham, MA, USA; 23227), separated by 12% SDS-PAGE electrophoresis and transferred to polyvinylidene fluoride (PVDF) membranes, as described previously (40). The membranes were blocked with 5% defatted milk for 1 hr, and then immunoblotted with anti-CD147 (Boster, California, USA; 1:500), anti-Beclin-1 (Boster, California, USA; 1:1000), and anti-GAPDH (Boster, California, USA; 1:1000) over night at 4 ^°^C. The membranes were washed, and then incubated with specific secondary antibodies. Finally, the blots were visualized using chemiluminescence (ECL; Forevergen Biosciences Center, Guangzhou, China).

**Figure 1:**
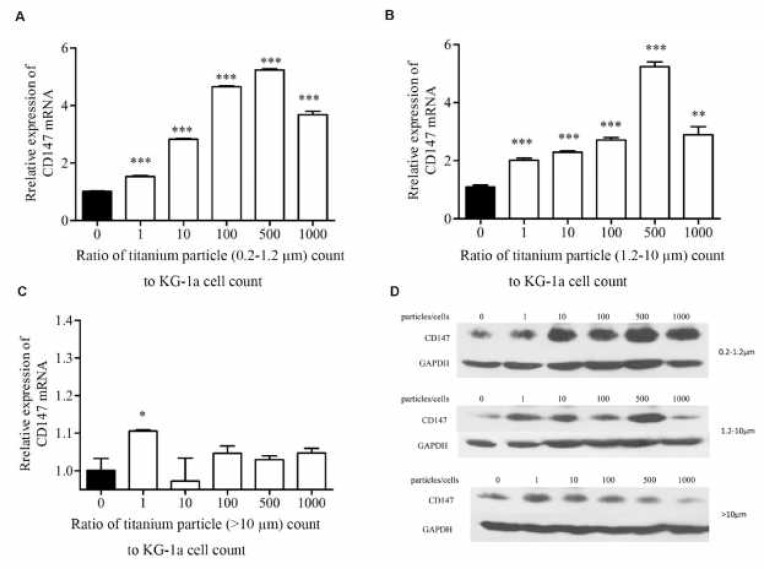
Effects of titanium particles on mRNA and protein level of CD147. QPCR showed the expression of CD147 mRNA in KG-1a cells co-cultured with 0.2–1.2 µm (A), 1.2-10 µm(B) and >10 µm (C) titanium particles; the protein level of CD147 in KG-1a cells co-cultured with 0.2–1.2, 1.2-10 and >10µm titanium particles was detected by western blot (D). *P<0.05; **P<0.01; ***P<0.001

**Figure 2:**
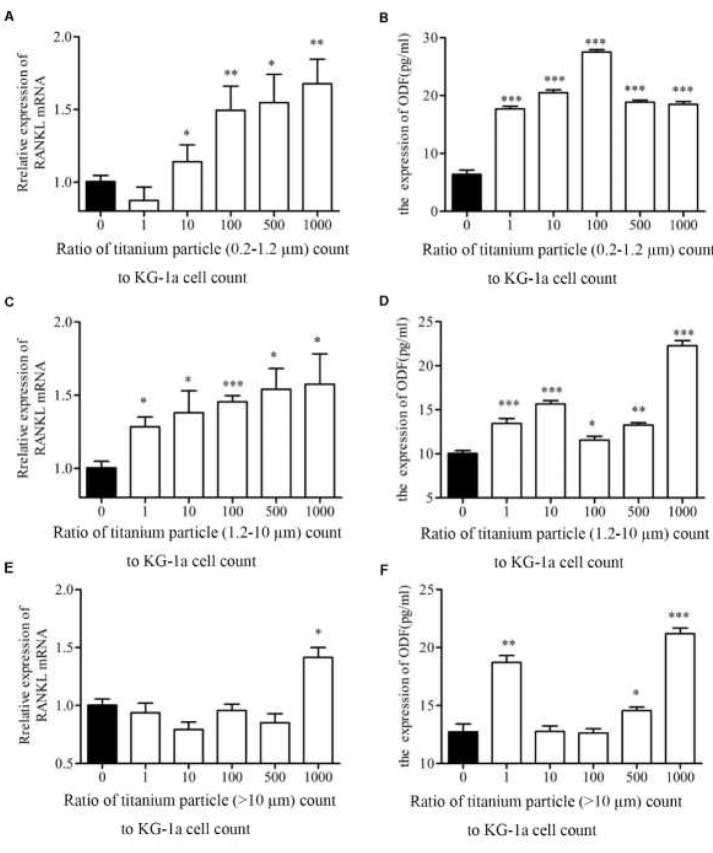
Effects of titanium particles on mRNA and soluble protein expression of RANKL. The mRNA of RANKL and soluble RANKL in KG-1a cells co-cultured with 0.2–1.2 µm (A, B), 1.2-10 µm (C, D) and >10 µm (E, F) titanium particles were detected by qPCR and ELISA respectively. *P<0.05; **P<0.01; ***P<0.001

**Figure 3:**
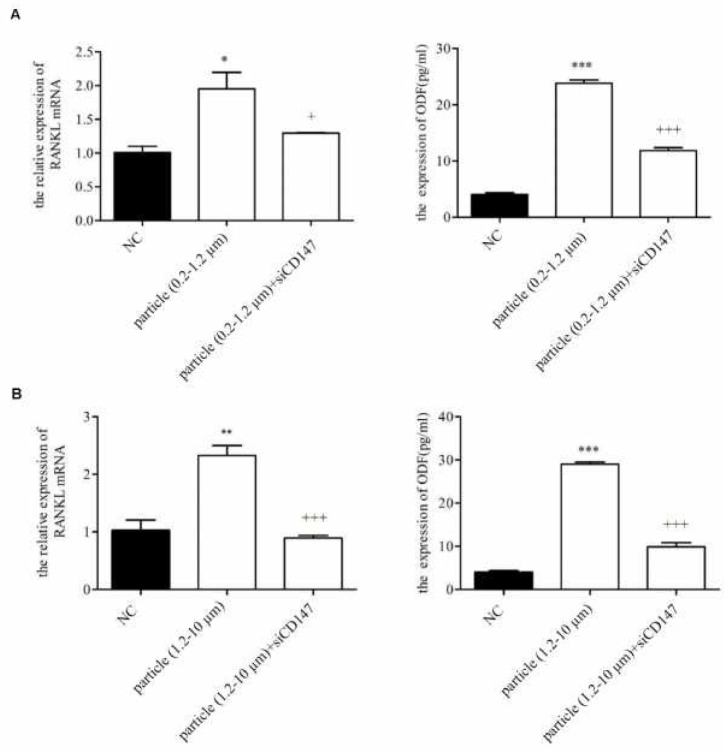
Effects of CD147 siRNA on mRNA and soluble protein expression of RANKL. The mRNA and soluble protein expression levels of RANKL in KG-1a cells co-cultured with CD147 siRNA and 0.2–1.2 µm (A), 1.2-10 µm (B) were detected by qPCR and ELISA respectively. NC: Normal control. *P<0.05; **P<0.01; ***P<0.001. +*P<*0.05; ++*P<*0.01; +++*P<*0.001

**Figure 4:**
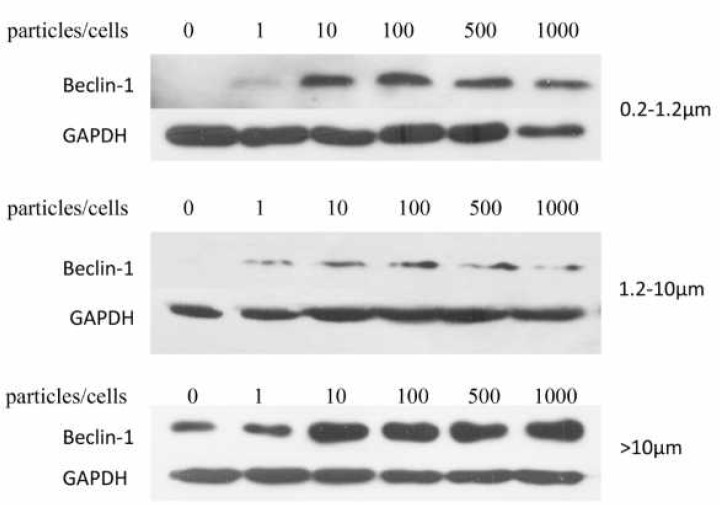
Effects of titanium particles on Beclin-1 protein expression. Protein levels of Beclin-1 in KG-1a cells co-cultured with 0.2–1.2 µm, 1.2-10 µm and >10 µm titanium particles were detected by western blot

**Figure 5:**
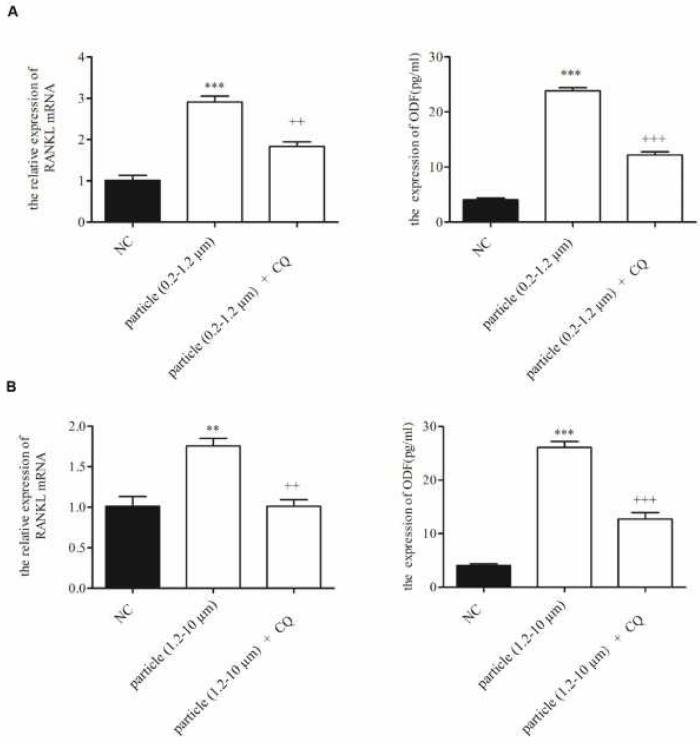
Effects of autophagy inhibitor CQ on mRNA and soluble protein expression of RANKL. The mRNA and soluble protein expression levels of RANKL in KG-1a cells cotreated with autophagy inhibitor CQ and 0.2–1.2 µm (A), 1.2-10 µm (B) were detected by qPCR and ELISA respectively. NC: Normal control. *P<0.05; **P<0.01; ***P<0.001. +*P<*0.05; ++*P<*0.01; +++*P<*0.001

**Figure 6:**
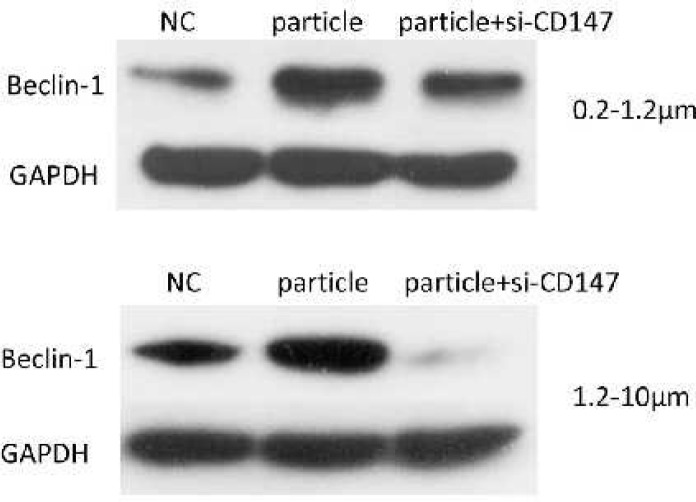
Effects of CD147 siRNA on Beclin-1 protein expression. Protein levels of Beclin-1 in KG-1a cells co-cultured with CD147 siRNA and 0.2–1.2 µm, 1.2-10 µm titanium particles were detected by Western blot

***Enzyme-linked immunosorbent assay (ELISA)***

The RANKL levels in the supernatants were detected using Human TNFSF11 ELISA Kit (Abcam, Cambridge, MA, USA; ab100749). The assay was performed as described previously (41). All procedures were performed according to the instructions.

After coating, plates were sequentially washed with PBST buffer and blocked with 1% BSA and incubated for 1 hr at 37 ^°^C. Then, anti-ODF and HRP-conjugated antibody were sequentially added and incubated for 1 hr at 37 ^°^C. The detection was achieved by adding chromogenic substrate, 3,3’,5,5’-tetramethylbenzidine (TMB). Absorbance was measured at 450 nm with an EnSpire multimode plate reader (Perkin Elmer, Waltham, Massachusetts).

***Statistical analysis***

All experiments were repeated at least three times. Data are presented as means±SD. Student’s t-test (unpaired, two-tailed) analysis were carried out to evaluate differences between groups. *P*<0.05 was considered statistically significant.

## Results

***Wear particles increase the expression of CD147***

CD147 has been reported to play an important role in breast cancer-induced osteolytic lesions. To assess whether CD147 expression was altered by titanium particles in macrophages, KG-1a cells were co-cultured with 0.2–1.2 µm, 1.2-10 µm and bigger than 10 µm (>10 µm) titanium particles for 24 hr, KG-1a cells were seeded in 6-well plates, 5 million cells per well, three ranges of particles were added to the corresponding well, the ratio of particle count to cell count was 1000:1, 500:1, 100:1, 10:1, 1:1, 0:1, respectively. The mRNA and protein expression levels of CD147 were detected with qPCR and Western blot. As shown in Figure 1A, B and C, the mRNA of CD147 was significantly increased in the cells co-cultured with 0.2–1.2 µm and 1.2-10 µm particles, and showed dose-dependent increase with rising ratios; while >10 µm particles was slightly increased at 1:1 ratio compared with the control, and no significant increase in CD147 mRNA for any of the other treatment groups. The protein of CD147 was dramatically elevated when the cells were incubated with 0.2–1.2 µm and 1.2-10 µm particles (Figure 1D). The western blot results implied that titanium particles sized 0.2–1.2 µm and 1.2-10 µm induced a dose-dependent increase of CD147 protein level with rising ratios. Nevertheless, >10 µm particles could not be able to significantly increase the CD147 protein level at different ratios (Figure 1D). These results indicated that titanium particles smaller than 10 µm were able to greatly increase the expression level of CD147 protein. 

***Down-regulation of CD147 suppressed wear particle-induced osteoclastogenesis***

RANKL plays a key role in osteoclast development, thus we assessed whether titanium particles altered the expression of RANKL in macrophages. Under treatment of titanium particles, the mRNA of RANKL in KG-1a cells were detected with qRCR. Our results suggested that the mRNA of RANKL was significantly up-regulated in the KG-1a cells treated with 0.2–1.2 µm particles at 10:1 or higher ratios, 1.2-10µm particles at 1:1 or higher ratios, and >10 µm particles at 1000:1 ratio (Figure 2A, C and E). To investigate whether titanium particles regulated RANKL secretion at the protein level, we evaluated soluble RANKL in supernatant of treated cells using ELISA. As shown in Figure 2B and D, at all ratios, titanium particles sized 0.2–1.2 µm and 1.2-10 µm were able to robustly increase the expression level of soluble RANKL. 4.3-fold and 2.2-fold up-regulation of soluble RANKL were observed in the cells, which were stimulated by 0.2–1.2 µm particles at 100:1 ratio and 1.2-10 µm particles at 1000:1 ratio, respectively. Titanium particles sized >10 µm could induce the expression of soluble RANKL only at ratios of 1:1(1.5-fold), 500:1(1.1-fold) and 1000:1(1.6-fold) (Figure 2F). These results showed different responses of macrophages to titanium particles of varying sizes, the titanium particles sized 0.2–1.2 µm and 1.2-10 µm exhibited more biological activity. Therefore, titanium particles sized 0.2–1.2 µm (at particle count to cell count ratio of 100:1) and 1.2-10 µm (at particle count to cell count ratio of 1000:1) were selected for subsequent experiments.

To determine whether CD147 was involved in titanium particle-induced osteoclastogenesis, CD147 siRNA (si-CD147) was used to down-regulate CD147 expression during titanium particles treatment. The mRNA and soluble protein expression levels of RANKL were detected with qPCR and ELISA. As shown in Figure 3A and B, the effect of titanium particles on RANKL was indeed CD147 dependent because in cells in which CD147 was down-regulated by siRNA, titanium particles failed to stimulate both mRNA and soluble protein expression of RANKL. 

***Autophagy mediated wear particle-induced osteoclastogenesis***

Previous studies have reported that metal particles could stimulate autophagy in osteoblasts. However, whether autophagy is involved in wear particle-induced osteoclastogenesis is unknown. Beclin-1 is fundamental for the formation of PI3K complexes, and acts at the initiation stage of autophagy. In the present study, we detected the protein expression levels of Beclin-1 in titanium particles treated KG-1a cells, and Beclin-1/GAPDH ratio was used as the indicator of autophagy. As shown in Figure 4, 0.2–1.2 µm and 1.2-10 µm titanium particles were able to robustly elevate the levels of Beclin-1. In >10 µm titanium particles treated cells, an increase of Beclin-1 was also observed, but showed no significant increase at 10:1 or higher ratios. Thus, titanium particles sized 0.2–1.2 µm (at particle count to cell count ratio of 100:1 ratio) and 1.2-10 µm (at particle count to cell count ratio of 100:1 ratio) were selected for subsequent experiments.

To investigate whether autophagy was required for titanium particle-induced osteoclastogenesis, qPCR and ELISA were performed to analyze the cells cotreated with titanium particles and autophagy inhibitor CQ. As shown in Figure 5, the up-regulation of RANKL mRNA was attenuated when the cells were treated with CQ. In addition, CQ treatment significantly suppressed the titanium particle-induced up-regulation of soluble RANKL protein in supernatants. These results suggested that autophagy was activated in response to 0.2–1.2 µm and 1.2-10 µm titanium particles, and involved in wear particle-induced osteoclastogenesis.

***Down-regulation of CD147 suppressed wear particle-induced autophagy***

Previous studies have suggested that CD147 was involved in starvation-induced autophagy in PC-3 cells. Thus, we sought to determine whether CD147 was essential for particle-induced autophagy in macrophages, the levels of Beclin-1 in siRNA and titanium particles co-treatment cells were detected via western blot. As shown in Figure 6, suppression of CD147 significantly attenuated titanium particle-induced Beclin-1 expression. The results indicated wear particle-induced autophagy was mediated by CD147.

## Discussion

Artificial joint replacement is considered as an effective treatment of end-stage joint diseases. However, wear particle-induced PIO and subsequent aseptic loosening remain the major cause of failed arthroplasty. Wear particles released from the bone-prosthesis interface are predominately phagocytosed by macrophages and alter the expression of genes in these cells, leading to the differentiation of macrophage precursors into giant cells or osteoclasts (42). RANKL regulates the osteoclast differentiation, the survival and function of mature osteoclasts. Thus, the level of RANKL is used as an indicator of how osteoclast differentiation can proceed (43). CD147 is a transmembrane glycoprotein and considered to function as an extracellular matrix metalloproteinase inducer. In the present study, we demonstrated that the size of titanium particles is a critical factor in macrophage activation. 0.2–1.2 µm and 1.2-10 µm titanium particles could increase the expression of CD147 and RANKL in KG-1a cells, inducing osteoclast differentiation. These results represented a slight reduction in the size of the most reactive particles compared with earlier studies, they were still within the phagocytosable size range identified previously to be the most biologically active (0.3-10 µm)(44). The largest (>10µm) particles failed to stimulate the cells to produce CD147 and RANKL, which may be explained by the macrophages inability to phagocytose the particles since they were too large. Furthermore, we showed that RANKL is a gene downstream of CD147. Knockdown of CD147 by specific siRNA mitigated the effects of titanium particles on expression of RANKL. These results suggested that titanium particles induce CD147, which may regulate RANKL, and thus promote osteoclast differentiation. 

Autophagy is an evolutionarily conserved process, which is responsible for degrading and recycling protein aggregates, oxidized lipids, damaged organelles and intracellular pathogens. Factors that induce cellular stress, such as absence of nutrient, deficiency of oxygen and accumulation of oxidative stress, can activate autophagy. During initiation stage of autophagy, phagophore is formed, occurring with the formation of two multiprotein complexes: ULK1 (unc51-like autophagy activating kinase 1) protein kinase complex and the PI3KC3-C1 (class III phosphatidylinositol 3-kinase complex I) lipid kinase complex (45). Beclin-1 is fundamental for the formation of PI3K complexes and important for the initiation of autophagy (46). Numerous evidences suggests that nanomaterials can induce autophagy and inflammatory response in immune cells (47-49). Nanomaterials phagocytosed are perceived as foreign or aberrant by cells, then autophagy is activated to try to degrade foreign substances. Recently, it has been reported that an increase in autophagy occurs during osteoclastogenesis, and pharmacological and genetic inhibition of autophagy reduces osteoclastogenesis and bone resorption in mice (32). In this study, we showed that 0.2–1.2 µm and 1.2-10 µm titanium particles could elevate the levels of Beclin-1 to promote the formation of autophagosome. Moreover, with the autophagy inhibitor CQ, we uncovered that autophagy mediates the up-regulation of RANKL induced by titanium particles. These results implied 0.2–1.2 µm and 1.2-10 µm titanium particles could be phagocytosed by macrophages, and activate autophagy, leading to up-regulation of RANKL. The largest (>10 µm) particles showed more biocompatible, since they are too large to be phagocytosed.

CD147 is highly expressed on the cell surface of most of cancer cells, and promotes tumor invasion, metastasis and growth. Several researches show that CD147 is associated with autophagy in epithelial ovarian cancer cells (50), prostate cancer cells (51) and hepatocellular carcinoma (37). In present study, with the siRNA against CD147, we revealed that CD147 mediate the wear particle-induced autophagy.

## Conclusions

The present study suggested that 0.2-1.2 µm and 1.2-10 µm titanium particles showed more biologically activity than >10 µm particles, and CD147 played a key role in autophagy and wear particle-induced osteoclastogenesis. Our results demonstrated that 0.2-1.2 µm and 1.2-10 µm titanium particles could stimulate the expression of CD147 to enhance autophagy, promoting osteoclastogenesis. More importantly, down-regulation of CD147 resulted in the significant decrease of autophagy and soluble RANKL protein in supernatants that were induced by 0.2-1.2 µm and 1.2-10 µm titanium particles. Our ﬁndings suggested a possible mechanism underlying wear debris-induced osteolysis and identiﬁed CD147 as a potential therapeutic target in aseptic loosening.
